# Examining the Effectiveness of the Fathers and Babies Intervention: A Pilot Study

**DOI:** 10.3389/fpsyg.2021.668284

**Published:** 2021-07-15

**Authors:** S. Darius Tandon, Jaime Hamil, Emma E. Gier, Craig F. Garfield

**Affiliations:** ^1^Center for Community Health, Northwestern Feinberg School of Medicine, Institute of Public Health and Medicine, Chicago, IL, United States; ^2^Department of Medical Social Sciences, Northwestern Feinberg School of Medicine, Chicago, IL, United States; ^3^Family and Child Health Innovations Program, Department of Pediatrics, Lurie Children’s Hospital of Chicago, Chicago, IL, United States

**Keywords:** intervention, home visiting, cognitive-behavioral therapy, paternal depression, maternal depression, behavioral technology

## Abstract

There is increasing recognition of the importance of addressing the mental health of fathers, including during the perinatal period. Fathers exhibiting mental health concerns during the perinatal period are at heightened risk for future negative mental health outcomes and are less likely to engage in nurturing relationships with their children, leading to a sequalae of negative child outcomes during infancy and into adolescence. Although interventions have been developed for perinatal fathers, they typically do not focus directly on addressing paternal mental health. To fill this gap, we developed the Fathers and Babies intervention to be delivered to perinatal fathers whose partners (mothers) were enrolled in home visiting programs. A pre-post longitudinal study was conducted in which 30 father-mother dyads were recruited from home visiting programs. Fathers received the 12-session Fathers and Babies intervention while the mother concurrently received the Mothers and Babies intervention delivered to her by a home visitor. Baseline, 3- and 6-month self-report surveys were conducted with both fathers and mothers. Fathers and mothers had statistically significant decreases in perceived stress between baseline and both follow-up time points, with moderate effect sizes generated for both sexes. No statistically significant differences were found for depressive symptoms, anxiety symptoms, or perceived partner support, although we found small effects for reductions in depressive symptoms among fathers, as well as increases in the percentage of fathers and mothers who reported high levels of emotional and instrumental support post-intervention. While preliminary, these findings suggest the potential for Fathers and Babies to positively impact the mental health of fathers in the perinatal period, and also signal the viability of home visiting as a setting for delivering this intervention. Future research should employ a comparison group to generate stronger evidence of intervention effectiveness and include measurement of dyadic relationships and paternal parenting practices.

## Introduction

Gradually over the past few decades, attention is being paid to paternal mental health and its impact on fathers and their children. Prevalence rates estimate that 5–13% of fathers will experience depression during their partner’s pregnancy and the first year postpartum (Paulson and Bazemore, 2010; Cameron et al., 2016; Pace et al., 2016), with a recent meta-analysis indicating that prior mental illness and paternal unemployment were the strongest predictors of paternal depression, with financial instability, limited social support, and low level of paternal education also associated (Ansari et al., 2021). Two meta-analyses have also highlighted positive, moderate correlations between paternal depression and maternal depression (Paulson and Bazemore, 2010; Thiel et al., 2020). The transition into fatherhood itself has been associated with an increase in depressive symptoms of as much as 68% in the first 5 years after the birth of the child (Garfield et al., 2014). A systematic review of paternal anxiety during the perinatal period found that between 2 and 18% of fathers experience anxiety during their partner’s pregnancy or in the first year postpartum (Leach et al., 2016), with a separate meta-analysis noting maternal depression, marital distress, and parental stress as having the strongest associations with paternal anxiety (Chhabra et al., 2020).

Paternal depression and anxiety have both been associated with decreases in positive father-child interactions and attachment (Wilson and Durbin, 2010; Davis et al., 2011; Vreeswijk et al., 2014; Nath et al., 2015). Poor paternal mental health has also been associated with delayed neuromuscular maturation at 6 months and increased negative interactions (corporal punishment) during infancy (Davis et al., 2011; Sethna et al., 2017). Beyond infancy, evidence of paternal depression in early fatherhood is a predictor of emotional and behavioral issues when the child is 4–5 years of age (Fletcher et al., 2011) and poor child language development (Paulson et al., 2009). Residing with fathers who exhibit depressive symptoms has also been associated with increased rates of emotional and behavioral problems among school-aged children and adolescents (Weitzman et al., 2011; Reeb et al., 2015). Paternal mood symptoms and disorders themselves are independently associated with child emotional and behavioral problems after adjustment for maternal psychological distress, predicting child emotional symptoms (Flouri et al., 2019), psychiatric disorders (Ramchandani et al., 2008), adolescent depressive symptoms (Lewis et al., 2017) and a range of problem behaviors, including conduct disorder and hyperactivity (Ramchandani et al., 2008; Flouri et al., 2019). Maternal depression, however, is an important mediator of the relationship between paternal depression and child behavior (Gutierrez-Galve et al., 2015), and children with two depressed parents are at particularly high long-term risk for mood disorders (Havinga et al., 2017).

Unfortunately, there has been limited attention placed on developing interventions specifically focused on addressing paternal mental health in the perinatal period. A systematic review conducted by Lee et al. (2018) found 19 interventions for fathers—delivered in the United States—during the perinatal period that had been tested using experimental or quasi-experimental designs. These interventions focused on general childbirth education and infant care, co-parenting skills, or case management with only four examining mental health outcomes (Diemer, 1997; Feinberg and Kan, 2008; Field et al., 2008; Salman-Engin et al., 2017). Of the four interventions that assessed paternal mental health, improvements in mental health were found only by Field et al. (2008) who generated reductions in paternal depressive symptoms via an intervention that taught fathers how to provide massages for their partner aimed at reducing pain and improving dyadic relationship quality.

Home visiting (HV) is a service delivery strategy that connects expectant parents and parents with young children with a designated supportive individual who may be a professional (e.g., nurse, social worker) or paraprofessional. There are 21 evidence-based HV models that have demonstrated positive impact on one or more maternal and child health outcomes using rigorous research designs (Office of Planning, Research and Evaluation, 2020). These HV models are voluntary and provide services in the family’s home, with models typically focusing on discussion of infant and young child development, linkages to prenatal and pediatric care, preparation for childbirth, and referrals to external providers to address physical or psychosocial risks.

While HV programs typically focus on the mother as client, recent years have seen considerable emphasis placed on including and engaging fathers in HV services. Fathers have reported that they view HV programs as trusted sources of information and value the services and information provided by HV (Child and Family Research Partnership, 2013). Evidence-based HV models vary in their approaches to engaging fathers through their service delivery, with most models focusing their efforts on promoting responsible fatherhood (Sandstrom et al., 2015). Fathers who are engaged in HV services alongside their partner have shown increased knowledge of child development, more responsive parenting practices, and greater connection with education, employment, and other community resources (Sandstrom et al., 2015). However, we are unaware of previous attempts to focus directly on addressing the mental health needs of fathers who engage in HV services alongside their partners.

This study’s overall aim was to develop and pilot test an intervention for fathers whose partners were enrolled in HV with the goals to improve paternal mental health and to help fathers support the mental health of their partner. FAB was designed to be delivered concurrently with the Mothers and Babies (MB) intervention—an evidence-based intervention based on principles of cognitive-behavioral therapy and attachment theory that has been found to be efficacious in preventing the onset of postpartum depression and reducing depressive symptoms via multiple randomized controlled trials (Muñoz et al., 2007; Le et al., 2011; Tandon et al., 2014, 2018; McFarlane et al., 2017), including several conducted in the context of HV (Tandon et al., 2014, 2018; McFarlane et al., 2017). A major goal of cognitive-behavioral approaches to is the development of skills for managing negative emotions and mood, which are often deficient in both mothers and fathers exhibiting depressive symptoms and underlie a parent’s ability to engage in well-regulated and responsive parenting practices that promote parent-child interaction and children’s self-regulation. Thus, the cognitive-behavioral approaches found in maternal-focused interventions like MB were posited to have similar value in reducing mental health adversity among fathers. Additional details on MB can be found in Le et al. (2015), and a more detailed description of the partnership between our research team and HV stakeholders to develop FAB can be found in Hamil et al. (under review). This manuscript reports on the paternal and maternal mental health outcomes associated with our pilot testing of FAB among a diverse group of father/mother dyads enrolled in HV.

## Materials and Methods

### Study Design and Participants

We used a single group longitudinal pre-post design to evaluate study outcomes. Nine HV programs served as project partners and referral sites. These HV programs had been previously trained on MB and had prior experience delivering MB to perinatal women. HV programs participated in a training webinar with study investigators to review FAB implementation, study design and participant recruitment. We received 37 father-mother dyad referrals, of whom 30 (81%) were enrolled.

Participants were initially screened for eligibility criteria by their HV programs. Eligibility requirements included English speaking dyads (mothers and fathers); women (mothers) ≥18 years old enrolled in HV programs who were pregnant or had a child ≤12 months old, and men (fathers) ≥18 years. The dyad (mother and father) both had to agree to participate in the study to be eligible. Non-biological fathers or biological non-resident fathers were eligible given the diverse relationship and co-habitation statuses of families receiving HV services.

Baseline demographic data for pilot participants (30 fathers and 30 mothers) are located in Table 1. Mean age for fathers was 27.7 years, while mothers’ mean age was 26.5 years. Both fathers and mothers were nearly equally distributed across race/ethnicity (Black, Hispanic, White). Slightly less than half of the dyads were married or engaged. Eighty percent of dyads enrolled in the study after their child had been born and the mean age of these postnatal enrollees’ children at time of enrollment was 3 months. Nearly all participants had obtained a high school degree or higher and all fathers were employed, at least part-time at baseline.

**TABLE 1:** FAB pilot participants: baseline demographic characteristics.

Characteristics	Fathers	Mothers
	(*n* = 30)	(*n* = 30)
Age (Mean, SD)	27.7 (6.0)	26.5 (5.5)
**Race (N,%)**		
Black/African American	11 (37)	10 (33)
Hispanic/Latino	9 (30)	9 (30)
White/Caucasian	8 (27)	8 (27)
Other	2 (7)	3 (10)
Week’ Gestation (Mean, SD) among prenatal enrollees (*n* = 6)		30 (4.7)
Age in month of child (Mean, SD) among postnatal mothers (*n* = 24)		3 (2.7)
**Employment status (N,%)**		
Not currently working	0 (0)	18 (60)
Working part-time	5 (17)	7 (23)
Working full-time	25 (83)	5 (17)
**Educational attainment (N,%)**		
<High school degree	3 (10)	3 (10)
High school degree/GED	12 (40)	6 (20)
Some college or beyond	15 (50)	21 (70)
**Relationship Status (N,%)**		
Married	8 (27)	
Engaged	5 (17)	
Single	10 (33)	
Living with partner, not married/engaged	7 (23)	

### Fathers and Babies Intervention

**FIGURE 1:**
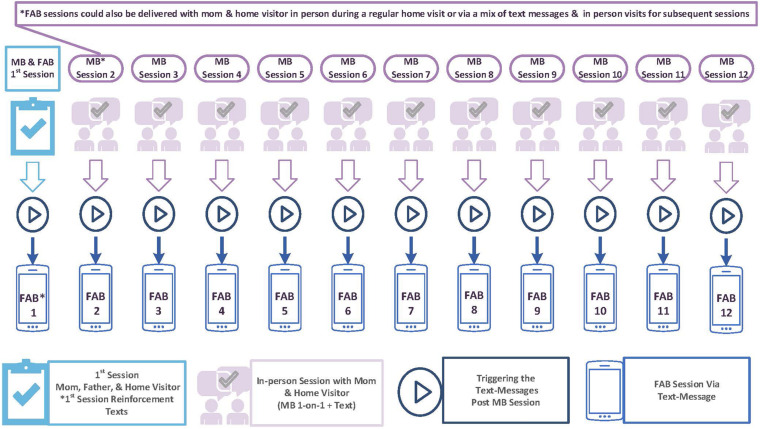
FAB and MB delivery.

The FAB intervention is described in additional detail in Hamil et al. (under review). It was designed to be delivered to partners of women who concurrently received the 12-session version of the MB postpartum depression preventive intervention from their HV program. Figure 1 depicts how FAB and MB are delivered. The initial FAB session was delivered in person or by phone by the home visitor working with the mother, and lasted 30 min on average. Subsequent sessions were delivered, in-person, via text message with embedded links to online content, or a mix of both in-person and text messages, depending on the preference and availability of the father. For fathers who received FAB in person, FAB content was delivered by the home visitor concurrent with delivery of MB to the mother using the FAB facilitator guide and FAB participant workbook. All fathers received the FAB workbook regardless of how they received the intervention to encourage them to use their workbooks to promote skill practice and to engage in conversations with their partner. The FAB facilitator guide provides instruction for delivering each of the 12 intervention sessions. Each session is broken into topics, with a script provided for each topic’s didactic content and interactive activities, as well a summary of key points that should be covered. The FAB workbook consists of a series of worksheets that corresponds with the FAB facilitator guide—for example, Worksheet 1.1 aligns with the first topic of the first intervention session. Worksheets are designed to be visually appealing and interactive in nature, with each worksheet allowing fathers to engage in intervention content being delivered. The last topic of each session asks fathers to engage in a “personal project” that promotes the practice of one or more skills that were discussed in the session.

When a father did not receive his FAB session in person, the FAB session was “triggered” when the home visitor delivered a MB session to the mother. Specifically, after the mother received her MB session, the home visitor documented MB session completion in our HealthySMS platform (Aguilera et al., 2017)^1^ —a web-based platform designed to send health-related text messages. Home visitor documentation of a completed MB session set in place the deployment of a series of text messages to the father. Fathers received three to six text messages per FAB session over the course of 7 days. The initial text messages had embedded links to external worksheets, videos, and other content that delivered the core FAB content. Subsequent text messages focused on reinforcing skill practice, reminding fathers to conduct personal projects assigned in the curriculum, and promoting self-monitoring of one’s mood. An example of a text message in each of these three areas is provided in Table 2. Fathers did not receive more than one text message per 24-h period and messages were automated to be sent at different times during the day between 8 a.m. and 10 p.m. The three to six text messages delivered the same amount of session material (i.e., content in the text message itself and content found when clicking the embedded links) over the 7 days as one in person session (30 min). Mothers also received three supplemental text messages between each session to reinforce skill practice and remind them about their personal projects.

**TABLE 2:** Example of the FAB text messages.

Skill reinforcement	Personal project reminder	Self-monitoring
Session 1:	Session 3:	Session 6:
We can do activities, change our thoughts, and seek support to help us manage our stress. FAB will help you manage stress and help you support your partner. ***LINK: Worksheet 1.1***	Pleasant activities can be low cost, brief, and part of our daily routines. You can do Pleasant Activities by yourself, with your partner, and with your baby. Link: https://www.first5california.com/en-us/videos/keeping-kids-physically-active-can-be-simple-and-fun/	Have you noticed any harmful thoughts you have? Reply Y/N [Also tell us if you used one of the talking back strategies to reduce it.

FAB is a 12-session intervention with content that mirrors the cognitive-behavioral therapy and attachment content found in MB. There is an introductory module followed by three cognitive-behavioral therapy modules: (1) pleasant activities, (2) thoughts, and (3) contact with others. Table 3 briefly describes core content found in each FAB module. It also illustrates the core content found in each of the corresponding MB modules. The goal of concurrent delivery of FAB and MB was to have both the father and mother receive similar CBT-related content that will provide each of them with skills to improve their own mood and help them support their partner’s use of cognitive-behavioral and attachment skills to also improve their partner’s mood. For example, the third FAB session focused on encouraging fathers to identify pleasant activities to help alleviate their own stress that can be easily integrated into their daily lives and routines. This FAB session also encouraged fathers to support their partners’ efforts to engage in pleasant activities as a way of attempting to improve mom’s mental health as well. The FAB facilitator guide explicitly points out places where conversations are encouraged with one’s partner around the CBT and attachment content being delivered.

**TABLE 3:** Overview of Mothers and Babies (MB) and Fathers and Babies (FAB) content, by intervention module.

Intervention module	Sessions	MB content	FAB content
Introduction	1–2	Relationship between stress and mood	How stress affects father-baby interactions and the relationship with your partner
		How stress affects mother-baby interactions	Purpose and overview of FAB
		Purpose and overview of MB	Relationship between stress and mood
		Importance of noticing one’s mood and its triggers	Importance of noticing one’s mood and its triggers
		Introduction to Quick Mood Scale	Introduction to Quick Mood Scale
Pleasant Activities	3–5	Relationship between pleasant activities and mood	Relationship between pleasant activities and mood
		Brainstorm pleasant activities to do alone, with adults, and with children	Brainstorm pleasant activities to do alone, w/adults, and w/children
		Pleasant activities with child can promote maternal-child bonding	Pleasant activities with child can promote paternal-child bonding
		Identify pleasant activities for mother-child bonding across baby’s first year	Identify pleasant activities for father-child bonding across baby’s first year
		Overcome obstacles to mothers doing pleasant activities	Overcoming obstacles to fathers doing pleasant activities
			Strategies to support mother’s engagement in pleasant activities
Thoughts	6-8	Relationship between thoughts and mood	Relationship between thoughts and mood
		Helpful and unhelpful thoughts about being a mother	Helpful and unhelpful thoughts about being a father
		Ways unhelpful thoughts inhibit maternal responsiveness	Ways unhelpful thoughts inhibit paternal responsiveness
		Ways to change unhelpful thought patterns	Ways to change unhelpful thought patterns
		Goals for my future	Goals for my future and ways to support my partners’ goals
		Goals for my baby’s future, including understanding importance of maternal-child bonding	Goals for my baby’s future, including understanding importance of paternal-child bonding
Contact with others	9–12	Relationship between mood and contact with others	Relationship between mood and contact with others
		Identify supportive people in one’s life and the ways they provide support to me and my child	Identify supportive people for me, my child, and my partner
		Communication styles to help get needs met	Communication styles to help get needs met
		Role changes and how they can increase need for social support	Role changes in becoming a father
		Role changes impact on relationship with other children	Role changes and how they increase need for social support in both mothers and fathers
			Role changes impact on relationship with other children

### Data Collection

The Northwestern University Institutional Review Board approved all study procedures. Fathers and mothers completed three self-report assessments—at baseline, 3-month follow-up, and 6-month follow-up. Survey links were sent via Research Electronic Data Capture (REDCap) (Harris et al., 2009) or administered via telephone by a member of the research team for participants who did not choose to complete their survey online. Participants provided informed consent via REDCap or via telephone prior to starting their baseline assessment. Compensation was $20 for completing the baseline and 3-month follow-up survey and $35 for completing the 6-month follow-up survey. All participants also received a stipend of $5 dollars per month while receiving the intervention to help offset text messaging costs. Dosage data was obtained from HealthySMS. Home visitors used HealthySMS to document completion of a MB intervention session, which also deployed FAB content to the father via text message if the father was not present for an in-person intervention session. A session was considered complete for both MB and FAB when the home visitor documented MB session completion in HealthySMS.

Among the 30 fathers who enrolled in the study, 80% (24/30) and 57% (17/30) completed 3- and 6-month follow-up assessments, respectively. For mothers enrolled in the study, 90% (27/30) and 77% (23/30) completed 3- and 6-month follow-up assessments, respectively.

### Instruments

#### Beck Depression Inventory-II (BDI-II) (Beck et al., 1988)

The BDI-II was used to assess severity of depressive symptoms consistent with DSM-IV symptom criteria. The BDI-II is a 21-item survey that asks respondents to indicate on a 4-point scale ranging from 0 to 3 the extent to which they endorse different symptoms of depression over the past 2 weeks with higher scores indicating greater depression severity.

#### Generalized Anxiety Disorder 7-Item Scale (GAD-7) (Spitzer et al., 2006)

The GAD-7 is a 7-item survey that asks respondents to indicate on a 4-point scale the extent to which they endorse different symptoms of anxiety over the past 2 weeks with higher scores indicating greater anxiety symptoms.

#### Perceived Stress Scale 10-Item Scale (PSS-10) (Cohen and Williamson, 1988)

The PSS-10 is a 10-item survey that asks respondents to indicate on a 5-point scale the extent to which they appraised certain situations as stressful over the past month, with higher scores indicating greater perceived stress.

#### Social Support Effectiveness Questionnaire (SSE-Q) (Rini et al., 2011)

The SSE-Q is a 25-item survey that asks respondents to indicate the extent to which their partners provided different types of support in the past 3 months. The SSE-Q consists of subscales on task support, informational support, emotional support, and negative effects of support. For this study, we calculated a total social support score that summed these four subscales (range 0–80).

#### NIH Toolbox Instrumental Support and Emotional Support Survey (Cyranowski et al., 2013)

Each survey consists of 8 questions and asks respondents to indicate on a 5-point scale the extent to which they have received different types of instrumental and emotional support in the last month. Higher scores indicate greater support.

### Analysis

Descriptive data (mean, standard deviation, range) were generated for all demographic variables and study outcomes. To assess paternal and maternal outcomes on the BDI-II, GAD-7, PSS-10, and SSE-Q, we conducted a series of paired t-tests with Bonferroni correction for multiple comparisons. *T*-tests compared baseline scores on each outcome to 3-month follow-up scores, with separate *t*-tests conducted to examine changes between baseline and 6-month follow-up. We used a Cohen’s *d* statistic (Cohen, 1988) to indicate effect sizes for our BDI-II, GAD-7, and PSS-10 outcomes. We calculated the percentage of mothers and fathers who reported scores above the cutoff for elevated depressive symptoms (BDI-II > 13) who moved below the cutoff at the 3- and 6-month follow-up time points. Similar analyses were conducted examining the percentage of mothers and fathers reporting scores above the cutoff for moderate severity of anxiety symptoms (GAD-7 > 10) who moved below the cutoff at each follow-up assessment. For the Instrumental and Emotional Support scales, we calculated the percentage of respondents at each time point who scored one standard deviation or more above the normed mean score of 50 which is suggestive of high levels of support (Cyranowski et al., 2013).

## Results

### FAB Dosage

Fathers participating in the FAB intervention received an average of 7.7 sessions (4.5SD), with a range of sessions from 1 to 12. The mode number of sessions received was 12, with 14 of the 30 (47%) of participants receiving the full FAB intervention. Dosage was identical for mothers receiving MB, as receipt of a MB session was the trigger for a father to have received his FAB intervention content (Figure 1).

### Paternal Mental Health Outcomes

Fathers on average entered the study with baseline scores of 6.5 on the BDI-II and 4.1 on the GAD-7, which fall into the mild symptom range for each assessment. Perceived stress at baseline was 14.9, which is suggestive of moderate stress levels. We found symptom declines for each outcome between baseline and 3-month follow-up with small, additional, symptom decline occurring between the 3- and 6-month follow-ups. Statistically significant improvements in perceived stress were found when comparing baseline to 3-month follow-up and 6-month follow-up means; observed *d* scores were 0.48 and 0.66, respectively, at each follow-up timepoint, indicative of moderate effect sizes. No statistically significant differences were found when comparing baseline and follow-up anxiety or depressive symptom scores, although *d* scores indicate small effect sizes when examining the magnitude of change in depressive symptoms. Among the five fathers who entered the study with elevated depressive symptoms, only two remained in the elevated range at both the 3- and 6-month follow-ups. Only one father entered the study with moderate anxiety symptoms, with this father dropping below the cutoff for moderate symptoms at the 3-month follow-up but returning to the moderate range at the 6-month time point. Paternal mental health outcomes are summarized in Table 4.

**TABLE 4:** Paternal and maternal outcomes from FAB pilot study.

	Fathers^a^					Mothers^b^				
		
	Baseline	3-Month		6-Month		Baseline	3-Month		6-Month	
						
	Mean (SD)	Mean (SD)	*d*	Mean (SD)	*d*	Mean (SD)	Mean (SD)	*d*	Mean (SD)	*d*
Depressive symptoms	6.5 (6.6)	4.5 (4.6)	0.35	3.6 (5.1)	0.24	9.7 (8.1)	8.4 (9.5)	0.21	8.3 (8.4)	−0.02
Anxiety symptoms	4.1 (4.5)	3.2 (3.4)	0.20	2.8 (3.2)	0.13	6.8 (5.5)	6.2 (5.1)	0.11	6.3 (4.8)	−0.08
Perceived stress	14.9 (7.6)	12.6 (7.1)^c^	0.48	10.9 (9.1)^c^	0.66	20.4 (9.1)	16.9 (7.4)^c^	0.57	16.8 (6.8)^c^	0.47
Social support effectiveness	60.9 (14.3)	57.8 (12.6)		59.3 (13.7)		48.9 (11.6)	51.4 (14.5)		52.2 (12.2)	
High emotional support^d^	30%	24%		50%		22%	32%		22%	
High instrumental support^d^	27%	28%		38%		13%	29%		41%	

*^*a*^Sample size at baseline (n = 30), 3-month follow-up (n = 24), 6-month follow-up (n = 17).*

*^*b*^Sample size at baseline (n = 30), 3-month follow-up (n = 27), 6-month follow-up (n = 23).*

*^*c*^p < 0.05.*

*^*d*^Percentage of respondents one standard deviation or more above normed mean.*

### Maternal Mental Health Outcomes

Mothers on average entered the study with higher baseline scores on the BDI-II (9.7) and the GAD-7 (6.8), although these scores still fall into the mild symptom range. Perceived stress at baseline was 20.4, which also falls into the moderate stress range. Similar to fathers, statistically significant reductions in perceived stress were found when comparing baseline to 3- and 6-month follow-ups, with observed *d* scores (0.57, 0.47) indicating moderate effect sizes. No statistically significant differences were found when comparing baseline and follow-up depressive symptom or anxiety symptom scores, although a small effect (*d* = 0.21) was found in examining the magnitude of change in depressive symptoms between baseline and 3-month follow-up. There were seven mothers who entered the study with elevated depressive symptoms, with five of these mothers remaining in the elevated range at both the 3- and 6-month follow-ups. Four mothers entered the study with moderate anxiety symptoms, with two of these mothers remaining in this range at both follow-up time points. Maternal mental health outcomes are summarized in Table 4.

### Social Support Outcomes

Fathers reported greater perceived support from their partner than mothers at baseline on the SSE-Q, although fathers’ perceptions of partner support decreased slightly over time. Mothers reported increased levels of perceived support from their partners at both follow-up assessments compared to baseline, although these improvements were not statistically significant. Results from the NIH Toolbox Instrumental Support survey found increases in both the percentage of fathers and mothers who exhibited high levels of instrumental support, as defined by scores = one standard deviation above the normed mean. We found that 27% of fathers exhibiting high instrumental support at baseline compared to 38% at 6-month follow-up while 13% of mothers exhibited high instrumental support at baseline compared to 41% at 6-month follow-up. A similar pattern was found among fathers when examining emotional support, with the percentage of fathers exhibiting high emotional support increasing from 30 to 50% between baseline and 6-month follow-up. Paternal and maternal social support outcomes are summarized in Table 4.

## Discussion

This study developed and pilot tested FAB among a diverse group of fathers whose partners were enrolled in HV programs and concurrently received the evidence-based MB intervention. Results on the pilot study’s acceptability and feasibility are presented in Hamil et al. (under review), with this manuscript describing paternal and maternal mental health outcomes associated with the FAB pilot study. Consistent with previous research, we found that mothers exhibited greater anxiety and depressive symptomatology than fathers (Wee et al., 2011; Darwin et al., 2021). We found statistically significant decreases in perceived stress among both fathers and mothers with corresponding moderate effect sizes. We also found small effect sizes associated with the magnitude of depressive symptom reduction among fathers. Results related to social support were mixed. Our measure of perceived partner support did not elicit any changes over time; however, broader assessment of social support found increases among both males and females who reported high levels of emotional and instrumental support post-intervention.

Fathers experience unique stressors during the perinatal period. From pregnancy, through the child’s birth and into infancy, fathers have been noted to experience stress related to negative feelings about pregnancy, changes in roles and responsibilities, and feeling incompetent in childcare (Philpott et al., 2019). Stress during this time period can in turn contribute to adverse mental health outcomes for fathers themselves and for their children. As such, our findings that FAB was able to significantly reduce paternal stress is notable. A systematic review conducted by Philpott et al. (2019) found that among 11 studies that reported on the impact of stress on fathers in the perinatal period, eight studies reported an association between higher stress levels and paternal mental health concerns including depression and anxiety (Johnson and Baker, 2004; Gao et al., 2009; Mao et al., 2011; Kamalifard et al., 2014; Wee et al., 2015). Paternal stress during the perinatal period has also been associated with behavioral concerns among fathers’ offspring during infancy and into early childhood (Choi et al., 2018; Lee et al., 2018). A recent meta-analysis found that perceived stress was among the strongest predicators of paternal postpartum depression (Ansari et al., 2021), suggesting that efforts to reduce perceived stress among fathers in the prenatal and early postpartum period may prevent the onset of postpartum depression and reduce depressive symptoms among fathers in the postpartum period. This is consistent with findings from our FAB pilot which found declines in paternal perceived stress and paternal depressive symptoms at both follow-up time points.

We found little change in perceptions of partner support, although there was some evidence suggesting that both fathers and mothers perceived higher levels of emotional and instrumental support post-intervention. Although discussion of social support permeates the entire FAB and MB curricula, most discussions occur during the last three intervention sessions. It is possible that more intentional focus on social support may need to be incorporated into earlier intervention content, along with additional and more prescriptive language for fathers on how to provide different types of support to their partner. Fathers and mothers receiving FAB and MB, respectively, may also have needed more time to practice using the social support skills taught in the intervention and that improved perceptions of partner support may have been exhibited if data collection had extended beyond 6 months. Our dosage data also indicated that while nearly half of the dyads received the entire FAB and MB interventions, the mean number of sessions on average was less than 8, which roughly translates to receipt of two-thirds of the intervention. Thus, several dyads in our sample did not receive the social support content found in FAB/MB since that content was found in the final module that began with session 9.

HV programs are an ideal setting for interventions aimed at improving paternal and maternal health and well-being. HV services delivered in a family’s home can serve large numbers of families, including the ones most in need of mental health services and those who are challenging to reach. Father engagement has been a high priority for HV programs in recent years, with a growing set of promising practices emerging (Sandstrom et al., 2015). Many of these practices have been incorporated into FAB, including father-centric engagement strategies, tailored content specific to fathers’ needs and experiences, and flexible delivery via text messages with embedded links to intervention content.

FAB is also likely to be of interest to HV programs who are seeking approaches to address maternal depression among their clients. It is estimated that nearly half of HV clients experience major depression or elevated depressive symptoms (Ammerman et al., 2010; Michalopoulos et al., 2015). MB has been increasingly sought out by HV programs for this reason, given its strong evidence base in preventing onset of major depression and reducing depressive symptoms among HV clients (United States Preventive Services Task Force et al., 2019). In light of this evidence base, MB has been designated by the Health Resources and Services Administration’s MIECHV HV initiative as an “approved” referral for HV clients who are experiencing depressive symptoms or major depression. As HV programs have been trained on MB in recent years, many have inquired about the availability of services and supports for fathers, which was a catalyst for our developing FAB. Findings presented in this manuscript about FAB’s impact on paternal and maternal mental health, juxtaposed with strong acceptability and feasibility data (Hamil et al., under review), suggest that FAB will be viewed as a potentially impactful tool for engaging fathers in HV services and improving their mental health. Fathers’ participation in HV services have been shown to promote mothers’ HV program engagement (Eckenrode et al., 2000; Korfmacher et al., 2008), which may also incentivize HV programs’ adoption of FAB. There may also be benefits for the mental health of mothers enrolled in HV if their partner receives FAB. Paternal depression has been consistently associated with elevated maternal depression (Paulson and Bazemore, 2010; Thiel et al., 2020), and persistence of maternal postpartum depression has been shown to be directly influenced by the presence of depression in their partners (Vismara et al., 2016). Thus, implementation of FAB in conjunction with MB may allow HV programs to more effectively address maternal depression than through their usual services, or their usual services enhanced only with mental health interventions that are maternal-focused.

### Study Strengths and Limitations

This study is among the first to examine paternal mental health outcomes associated with receipt of an intervention for fathers in the perinatal period. Moreover, FAB is the first intervention to our knowledge that explicitly focuses on paternal mental health that has been implemented in the United States (Lee et al., 2018), although researchers in Australia (Mihelic et al., 2018) and Pakistan (Husain et al., 2021) have also developed mental health interventions for perinatal fathers. We recruited a sample of racially and ethnically diverse fathers, including non-biological partners or biological non-resident fathers, given the growing number of households with contemporary family structures like these (Child Trends, 2018). Fathers from diverse racial and ethnic backgrounds also exhibit higher rates of unemployment than their white counterparts (Bureau of Labor Statistics, 2020). Given that paternal unemployment has been shown to be the strongest predicator of paternal depression (Ansari et al., 2021), our inclusion of racially and ethnically diverse fathers in our pilot suggests that we are focusing on fathers at heightened risk for poor mental health outcomes. This study is also strengthened by integrating FAB into an existing service—HV—that is trusted by fathers (Sandstrom et al., 2015) and has great potential for scaling given the presence of HV across the United States.

There are important limitations to consider in interpreting our study findings. Our pilot did not include a comparison group, so it is possible that the improvements in mental health outcomes were associated with forces external to FAB. This is perhaps more likely for mothers who were simultaneously receiving HV services since these services may have helped to offset stressors via provision of different types of instrumental (e.g., diapers) and informational (e.g., knowledge of child development) support. We were only able to collect pre-birth depressive symptoms from 25% of enrolled fathers since the other 75% of fathers enrolled postnatally. Attrition among fathers at our follow-up time points was higher than among mothers, thereby limiting generalizability of our findings. This could attributed to the fact the mothers had pre-existing relationships with their HV program and, therefore, may have had a stronger relationship with them than the fathers by virtue of being the primary HV client. Given the limitations of our sample size, we did not conduct analyses examining potential dosage effects of FAB. It is possible that fathers who received more FAB content exhibited improved outcomes. Modest impact on paternal or maternal social support alluded to a possible dosage effect, as individuals who did not receive the full intervention would not have benefited from the social support module which is delivered at the end of the FAB intervention. It is also important to point out that in calculating dosage, fathers who had the series of FAB text messages for a session deployed to them were deemed to have received that session although there may have been variability in the amount of time spent by fathers reviewing intervention content. Finally, fathers entered the study with relatively mild depressive symptoms. While some maternal and paternal depressive symptoms are common in the perinatal period, fathers are more likely to present with irritability symptoms as well as alcohol and substance use (Walsh et al., 2020). Future research examining FAB intervention effects should consider using an assessment tool specifically designed to capture paternal depression symptoms that includes somatization and externalizing items that are likely to be more commonly endorsed among men (Psouni et al., 2017).

### Future Directions

Future testing of FAB’s impact on paternal and maternal mental health outcomes is needed using experimental designs to provide stronger evidence of the intervention’s effectiveness. This future research should continue to examine mental health and father engagement outcomes, but also expand its focus to examine other outcomes related to dyadic relationships. Longitudinal follow-up beyond 6 months could also help illuminate whether effects on paternal and mental health seen in this pilot are sustained over time and whether other outcomes such as social support may exhibit improvements after intervention recipients are able to practice core cognitive-behavioral therapy skills related to expanding and more effectively activating one’s support network. Given the strong interest among HV programs in father engagement, future research should also examine whether participating in an intervention like FAB is associated with fathers’ engagement in other types of HV services. Data could also be collected from HV programs’ management information systems to ascertain potential impact of FAB on mothers’ program retention.

Subsequent trials should also consider selection criteria for FAB participants. For our pilot, we did not exclude dyads except if they had a child > 12 month old or did not feel comfortable receiving the intervention in English. Meta-analyses have identified risk factors most strongly associated with paternal (Ansari et al., 2021) and maternal (Guintivano et al., 2018) depression. For example, history of previous depression and relationship discord are two of the strongest predictors of both paternal and maternal depression. Although delivering of FAB and MB universally may be desirable to HV programs since it de-stigmatizes intervention participation, we may find greater evidence of intervention effectiveness if additional inclusion and exclusion criteria were added that would identify individuals at greatest risk for poor mental health outcomes in the perinatal period.

Results from this study also suggest that there may need to be revisions made to FAB content, and the manner in which the intervention is delivered, to heighten its impact. In light of our pilot findings and data related to the intervention’s feasibility and acceptability (Hamil et al., under review), we are refining intervention content in several ways, such as the inclusion of additional skills-based activities to improve perceptions of social support and tailoring content that model FAB skills across child development phases. We are also creating a welcome packet for fathers and adding more father-centric graphic design elements into the FAB curriculum as strategies for promoting greater engagement with intervention content.

HV programs are not the only setting where perinatal women present for services. Accordingly, as FAB is further refined and tested it is important to think about other settings in which the intervention could be implemented that could reach fathers such as primary care clinics and community health centers. There may also be settings where fathers are more easily reached such as employment or “re-entry” programs. While FAB is currently developed to be delivered concurrently with MB, it may be useful to also think of FAB as a free-standing intervention to reach a larger number of perinatal fathers who could from its content uncoupled from MB implementation.

## Conclusion

With the gradual increase in research to understand the contributions fathers make to families, national and international professional societies have called for greater focus on fathers and their mental health. Among these is the most recent American Academy of Pediatrics clinical report on fathers and pediatrics which highlights advances in understanding the role fathers play and the impact paternal depression may have on the family (Yogman et al., 2016). Similarly, HV programs are calling for improved services, along with evaluations of these services, as one way to support fathers and the needs of the entire family ecosystem. As more research becomes available on the association of paternal mental health and downstream child outcomes, programs such as FAB will be critical as they meet a growing need and can be disseminated at scale to serve a diverse population of men during the transition to fatherhood. At the heart of this work is the notion that the health and human services sector needs to be transformed to provide additional supports—especially as relates to fatherhood wellbeing and mental health. Doing so ensures that fathers are supported to the benefit of themselves, their partners and their children.

## Data Availability Statement

The raw data supporting the conclusions of this article will be made available by the authors, without undue reservation.

## Ethics Statement

The studies involving human participants were reviewed and approved by the Northwestern University School of Medicine Institutional Review Board. The patients/participants provided their written informed consent to participate in this study.

## Author Contributions

ST, JH, and CG were responsible for the design of the work. JH oversaw data collection. ST, JH, and EG were involved in data analysis. All authors were involved in interpretation of findings, drafting the article, critically reviewing the article, and have approved of the version that has been submitted.

## Conflict of Interest

The authors declare that the research was conducted in the absence of any commercial or financial relationships that could be construed as a potential conflict of interest.

## Footnotes

1 www.healthysms.org
